# Bariclip Removal and Conversion to Gastric Sleeve: A Case Report

**DOI:** 10.7759/cureus.71194

**Published:** 2024-10-10

**Authors:** Ricardo Xavier Cuellar-Tamez, Milton Alberto Muñoz Leija, Omar F Wong Rodríguez, Cristian Santiago Ramírez-López, José Patiño-Gallegos

**Affiliations:** 1 Surgery Department, Hospital Zambrano Hellion TecSalud, San Pedro Garza García, MEX; 2 General Surgery Department, Universidad de Monterrey, San Nicolas de los Garza, MEX

**Keywords:** bariatric surgery, bariatric surgery complications, gastric sleeve surgery, laparoscopic gastric sleeve gastrectomy, revisional bariatric surgery

## Abstract

Obesity has been recognized as the main pandemic of this century. Multiple treatments have been developed: the use of medications, exercise, diet, and surgery. Bariatric surgery is one of the treatments that has shown the greatest effectiveness. Several techniques have been developed, such as gastric sleeve (GS) and gastric bypass. One of the most recent is laparoscopic bariclip gastroplasty (LBCG). It is a procedure that involves placing a non-adjustable device parallel to the lesser curvature. It exerts a restrictive effect similar to GS. The main complications associated with this procedure include device migration and de novo gastroesophageal reflux disease (GERD). These are the main reasons for performing revision surgery and device removal. Weight regain is a rarely reported complication, and conversion to another procedure also has few reports. The objective of this study is to report the case of a female patient who presented weight regain as a complication and underwent conversion to GS in northeastern Mexico.

## Introduction

The obesity epidemic affects more than two billion people worldwide. Statistics show a significant increase in this condition since 1980, and it continues to rise [1]. Various treatments related to diet, exercise, and pharmacotherapy have been studied and developed; however, bariatric surgery has proven to be the most effective [2,3]. In recent years, different procedures have been explored, including Roux-en-Y gastric bypass (RYGB), sleeve gastrectomy (SG), adjustable gastric band, single anastomosis bypass, duodenal switch, and surgical implants such as the bariclip, among others [2,3].

Laparoscopic bariclip gastroplasty (LBCG) is one of the most recent treatments to gain popularity. It is a non-adjustable device placed vertically parallel to the lesser curvature of the stomach, producing a restrictive effect similar to that of SG [3,4]. However, like any procedure, it has its advantages and disadvantages. Advantages include its reversibility, reduced risk of staple line leakage, and fewer side effects compared to SG. The main disadvantages are associated with migration and erosion [4,5].

Few complications related to this device have been reported, with the primary issues being migration and a re-intervention rate of nearly 6% [5]. To the authors' knowledge, no cases of weight regain associated with this device have been reported. Therefore, the aim of this article is to present the case of a female patient who underwent this procedure and was successfully treated with conversion to SG in our hospital. Our case complies with the Surgical Case Report (SCARE) criteria [6].

## Case presentation

A 30-year-old female patient with a surgical history of bariclip placement one year ago in another country presented to our hospital with weight regain over the past few months. Denies gastrointestinal symptoms. Her BMI prior to bariclip placement was 32.8. At our hospital, she presented with a BMI of 36.9. Preoperative studies were conducted, including an endoscopy and barium swallow test, without presenting data on migration or erosion. It was decided to perform revision surgery with conversion to gastric sleeve (GS).

The procedure was conducted under general anesthesia, with the patient in the French position. The team was organized with the first surgeon in front of the patient, the second surgeon on the left, and the third assistant on the right. The pneumoperitoneum was established at 15 mmHg at the supraumbilical point using the Veress method. A 5 mm trocar was inserted at the supraumbilical midline. A diagnostic laparoscopy was performed without complications. Under direct visualization, the remaining ports were inserted: a 12 mm port in the right hypochondrium, a 12 mm port in the left hypochondrium, and a 5 mm left flank port.

**Figure 1:**
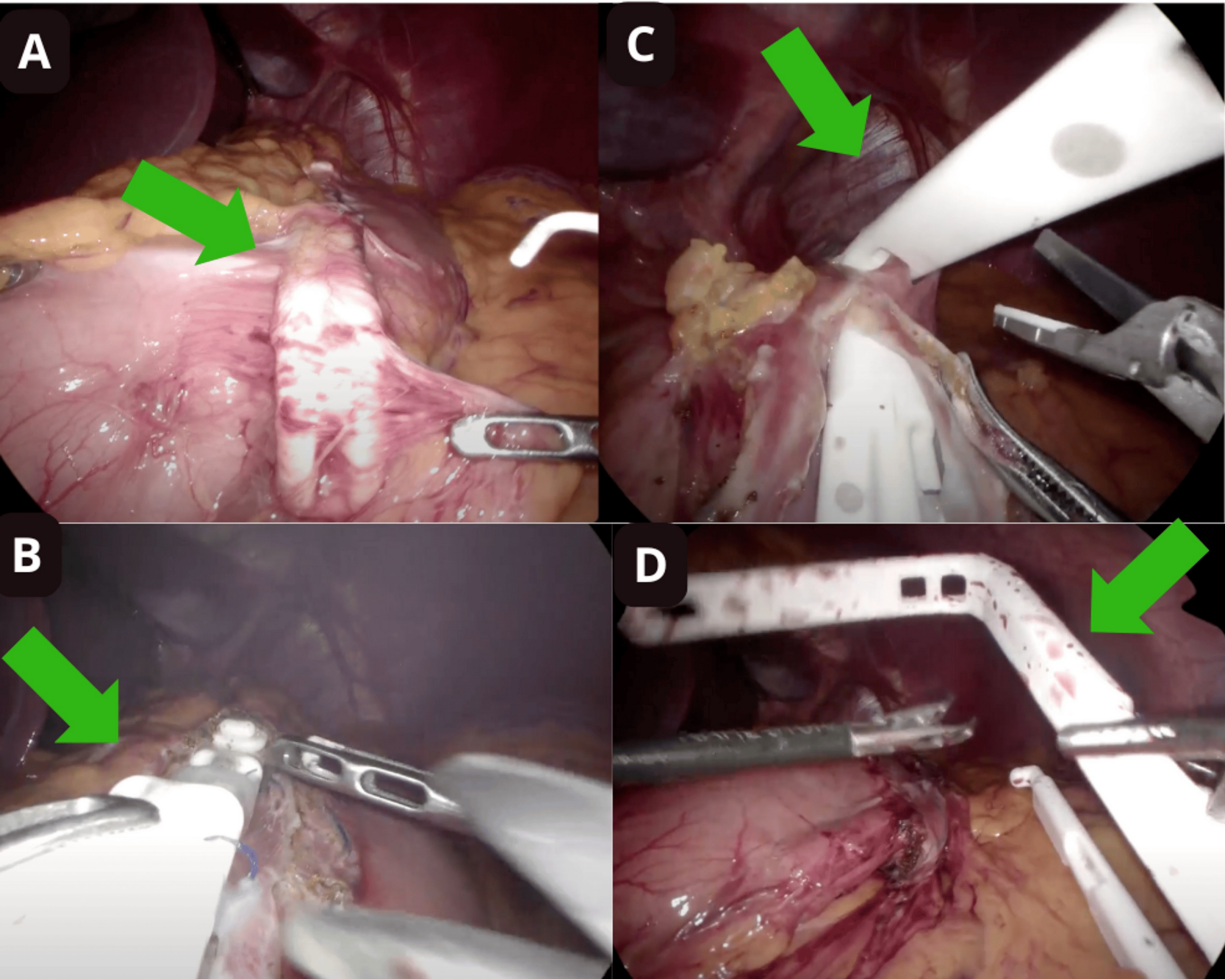
Laparoscopic view in the abdomen. (A) Bariclip in the major curvature of the stomach (green arrow). (B) Liberation of previous sutures of bariclip with ultrasonic energy (green arrow). (C) Liberation of bariclip with ultrasonic energy (green arrow). (D) Bariclip removed (green arrow).

Bariclip was identified in the major curvature of the stomach (Figure 1A).

Dissection began with the release of the bariclip capsule, and previous sutures on the stomach was removed with ultrasonic energy (Figures 1B, 1C). Hemostasis was confirmed, and the bariclip was removed (Figure 1D).

A conventional SG was performed. The first step was to sever all the short gastric vessels up to the fundus (along the entire greater curvature of the stomach). Subsequently, a 36-Fr orogastric tube was inserted 4 cm proximal to the pylorus of the stomach. Using the Echelon Flex stapler (Johnson & Johnson, USA), with two green cartridges followed by three black cartridges, the creation of the new stomach was performed. After the stapling was completed, the staple line was reinforced with Prolene 2-0 sutures.

**Figure 2:**
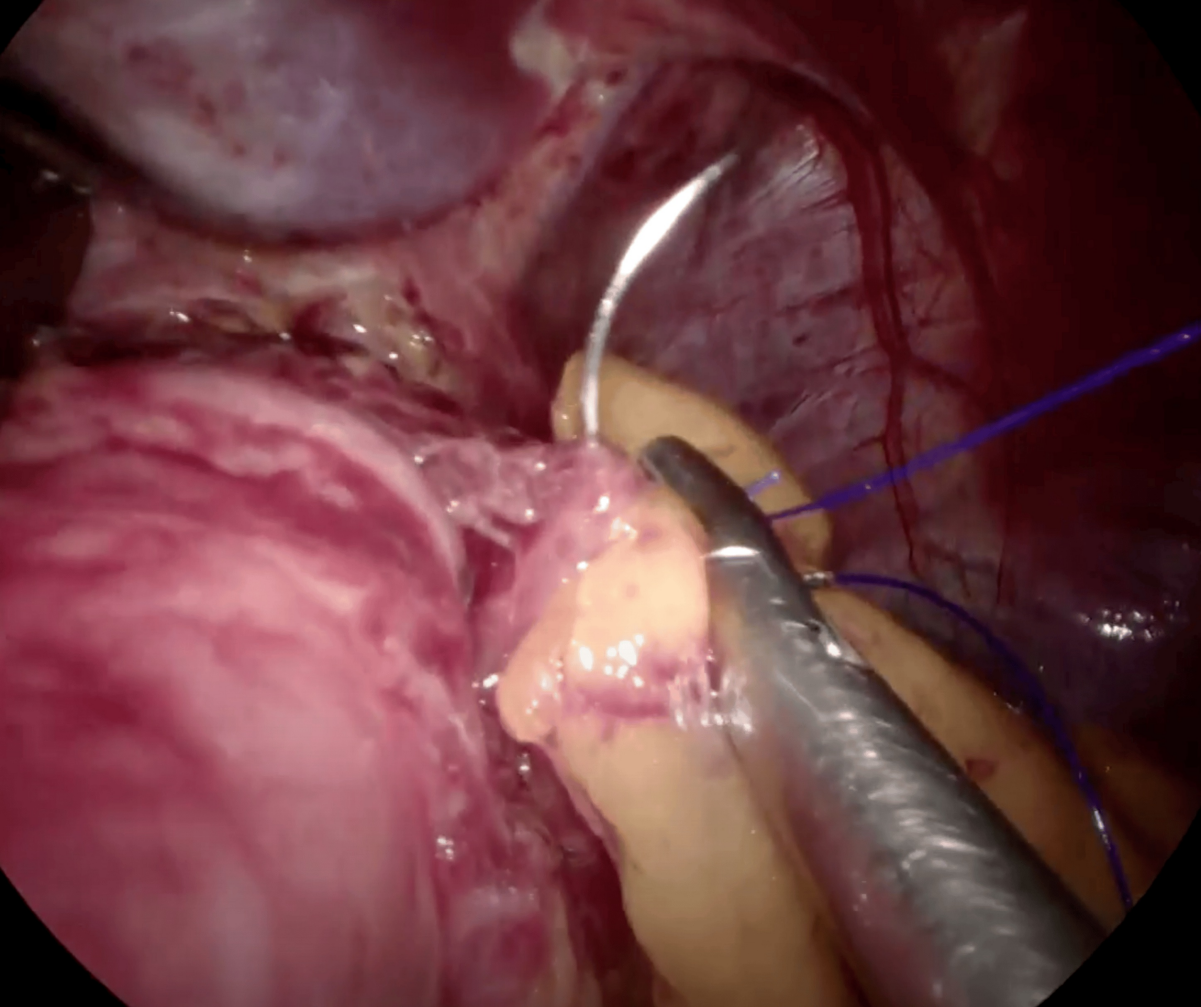
Gastric sleeve omentopexy with non-absorbable suture.

After verifying hemostasis, omentopexy was performed with Prolene 2-0 at the upper third of the new stomach (Figure 2).

The 12 mm supraumbilical port was closed with a non-absorbable suture using the Carter-Thomason device (Carter-Thomason CloseSure System; CooperSurgical Inc., Trumbull, USA). The skin was closed with a monofilament suture.

**Figure 3:**
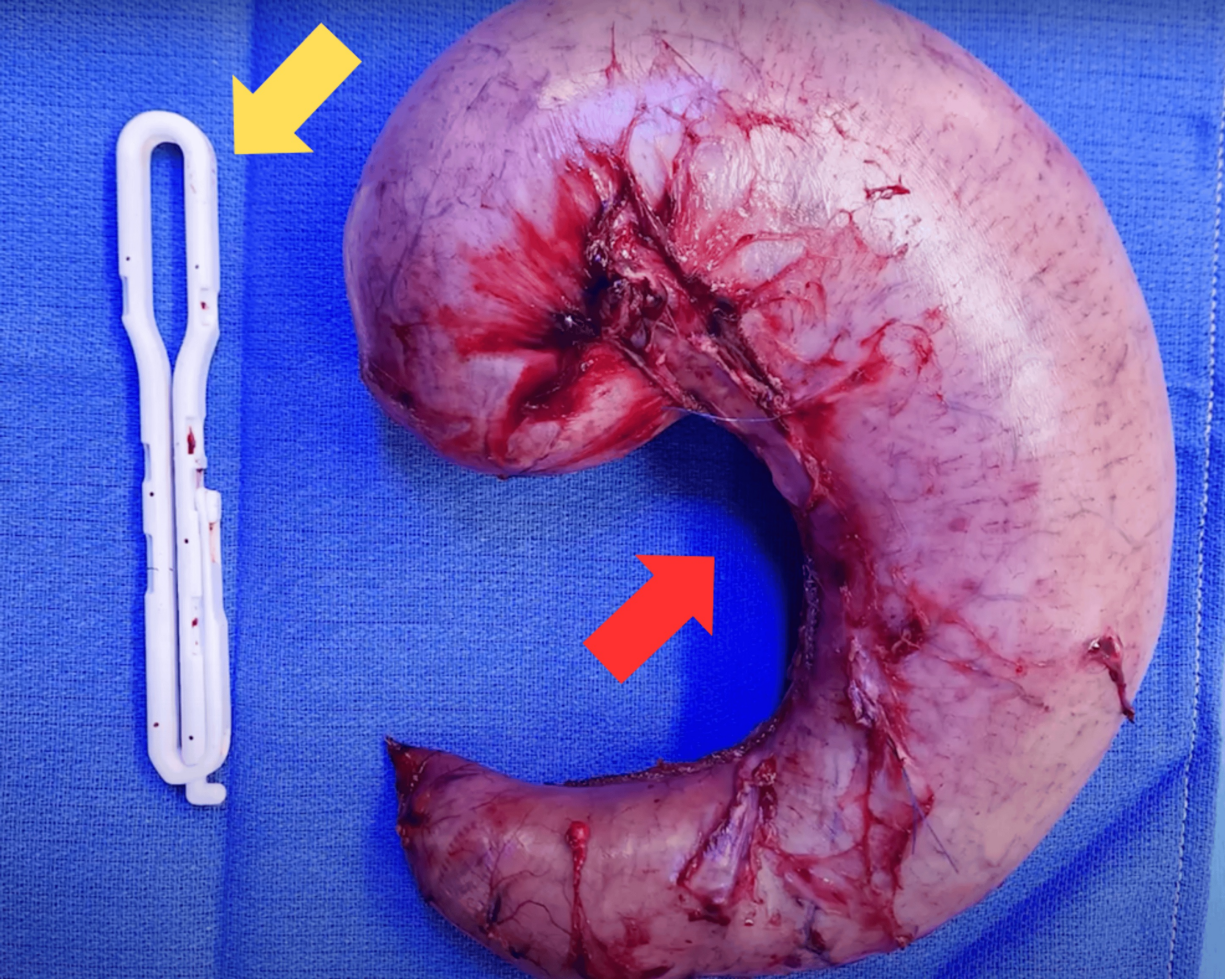
Bariclip removed (yellow arrow) and stomach removed (red arrow).

The surgical specimens (bariclip and the removed stomach) were sent to pathology (Figure 3).

**Figure 4:**
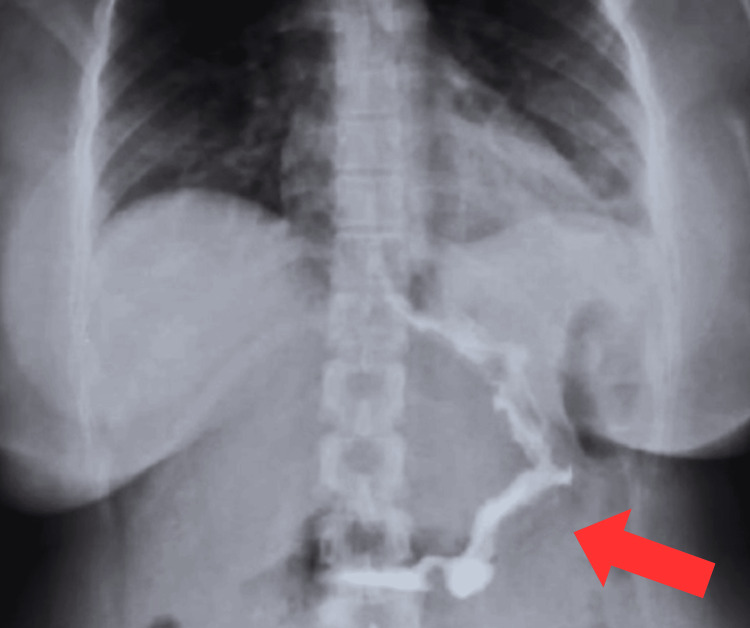
Post-surgical barium swallow test. New stomach with adequate liquid transit (red arrow).

Following our institutional postoperative follow-up protocol, a barium swallow test was performed 24 hours after surgery with adequate liquid transit (Figure 4).

At 48 hours postoperatively, the patient was discharged with adequate tolerance for liquids. After 18 months of follow-up, the patient had a BMI of 26.7 and a satisfactory postoperative outcome.

## Discussion

LBCG is one of the more recent bariatric procedures. It has been described in the literature as an alternative that offers improved quality of life and better outcomes with reduced post-surgical risks such as bleeding or leakage, in addition to being a reversible procedure that does not involve anastomosis and provides a restrictive mechanism [7].

Bashour from the Department of Surgery in Texas reported the first use of a gastric clip in 1985. This clip was made of relatively inflexible plastic and was placed in the upper third of the stomach. It functioned similarly to gastric bands. Over time, the clip did not yield good results as it did not produce adequate weight loss in patients, and high rates of complications such as erosion and gastric obstruction were reported. The technique was abandoned due to these issues. The failure was attributed to the type of material used and the anatomical site where it was placed [8-10]. However, in recent years, this technique was redesigned and now consists of a titanium capsule with silicone containing a lower opening with hinges [5].

Recent studies have evaluated both the benefits and complications in the short and long term. One study conducted in Italy assessed 50 patients who underwent LBCG. They reported major complications in 6% of cases, mainly slippage, and surgical re-intervention was required in three patients. One patient experienced lateral slippage post-imaging, and a second surgery was performed to reposition the clip. Two patients developed gastric obstruction with emesis approximately four months after clip placement. In these cases, the procedure performed was the removal of the device. Minor complications reported had a prevalence of 4%, including surgical site hematoma and de novo gastroesophageal reflux disease (GERD). Weight regain was not reported in any patient, and no conversions to other bariatric surgeries were performed (one patient had the clip replaced, and the other two had it removed) [5]. This contrasts with our case, where weight gain was a complication, and the surgical procedure involved clip removal and conversion to SG.

A study conducted in the United Arab Emirates in 2022 evaluated 43 patients who underwent LBCG. The reported complications included one patient with de novo GERD one year after the procedure. Two patients experienced slippage. In one patient, surgical intervention was performed to remove the clip, while the second patient received symptomatic treatment for nausea and pain due to minimal slippage. One patient experienced excessive weight loss after 14 months with the clip, leading to a decision to perform surgical removal [7].

A study conducted in our country compared different bariatric surgical techniques in 2024 [11]. A total of 49 patients were evaluated, comparing intragastric balloon, SG, RYGB, and LBCG. In the LBCG group, nine patients were evaluated. No complications were reported after 12 months of follow-up. Among the different groups, patients who underwent LBCG lost the most weight. In contrast, in our case, the complication was weight gain.

To the authors' knowledge, this is the first case reporting weight gain as a complication of LBCG in the literature. Further studies are needed to assess factors associated with this rare complication. Conversion to SG may be a viable option when such complications arise.

## Conclusions

Bariatric surgery is an effective and safe method for weight loss. The LBCG procedure presents a low complication rate, with slippage being the primary issue. Weight regain is a very uncommon complication of this procedure. Conversion to GS appears to be a good alternative for reversing this complication. Further studies are needed in the literature to evaluate the complications associated with the bariclip procedure and the solutions that can be offered to patients.

## References

[1] Afshin A, Forouzanfar MH, Reitsma MB (2017). Health effects of overweight and obesity in 195 countries over 25 years. N Engl J Med.

[2] Angrisani L, Santonicola A, Iovino P, Vitiello A, Zundel N, Buchwald H, Scopinaro N (2017). Bariatric surgery and endoluminal procedures: IFSO worldwide survey 2014. Obes Surg.

[3] Zerrweck C, Espinosa O (2020). Nuevas tecnologías y avances en terapias para la pérdida de peso. Rev Gastroenterol Mex.

[4] Jacobs M, Zundel N, Plasencia G, Rodriguez-Pumarol P, Gomez E, Leithead J 3rd (2017). A vertically placed clip for weight loss: a 39-month pilot study. Obes Surg.

[5] Gentileschi P, Campanelli M, Sensi B (2023). Safety and efficacy of laparoscopic vertical clip gastroplasty: early results of an Italian multicenter study. Obes Surg.

[6] Sohrabi C, Mathew G, Maria N, Kerwan A, Franchi T, Agha RA (2023). The SCARE 2023 guideline: updating consensus Surgical CAse REport (SCARE) guidelines. Int J Surg.

[7] Noel P, Layani L, Manos T (2022). The reflux and Bariclip: initial results and mechanism of action. J Clin Med.

[8] Bashour SB, Hill RW (1985). The gastro-clip gastroplasty: an alternative surgical procedure for the treatment of morbid obesity. Tex Med.

[9] Chao SH (2010). Gastric clipping for morbid obesity: the initial results of a clinical trial. World J Surg.

[10] Chang CG, Provost DA (2004). Gastro-Clip gastroplasty: a very long-term complication. Obes Surg.

[11] Myers EJA, Maldonado PDG (2024). Resultados comparativos de las técnicas de cirugía bariátrica para el tratamiento de la obesidad por un grupo quirúrgico en el Hospital Angeles Pedregal. Acta Med GA.

